# Shallow WGS of individual CTCs identifies actionable targets for informing treatment decisions in metastatic breast cancer

**DOI:** 10.1038/s41416-022-01962-9

**Published:** 2022-09-10

**Authors:** Daniel Fernandez-Garcia, Georgios Nteliopoulos, Robert K. Hastings, Amelia Rushton, Karen Page, Rebecca C. Allsopp, Bana Ambasager, Kelly Gleason, David S. Guttery, Simak Ali, R. Charles Coombes, Jacqueline A. Shaw

**Affiliations:** 1 Leicester Cancer Research Centre, Department of Genetics and Genome Biology, University of Leicester, Robert Kilpatrick Clinical Sciences Building, Leicester Royal Infirmary, Leicester, LE2 7LX UK; 2 Department of Surgery and Cancer, Imperial College London, Hammersmith Hospital Campus, Du Cane Road, London, W12 0NN UK

**Keywords:** Prognostic markers, Breast cancer

## Abstract

**Background:**

We report copy-number profiling by low-pass WGS (LP-WGS) in individual circulating tumour cells (CTCs) for guiding treatment in patients with metastatic breast cancer (MBC), comparing CTC results with mutations detected in circulating tumour DNA (ctDNA) in the same blood samples.

**Methods:**

Across 10 patients with MBC who were progressing at the time of blood sampling and that had >20 CTCs detected by CellSearch^®^, 63 single cells (50 CTCs and 13 WBCs) and 16 cell pools (8 CTC pools and 8 WBC pools) were recovered from peripheral blood by CellSearch^®^/DEPArray™ and sequenced with Ampli1 LowPass technology (Menarini Silicon Biosystems). Copy-number aberrations were identified using the MSBiosuite software platform, and results were compared with mutations detected in matched plasma cfDNA analysed by targeted next-generation sequencing using the Oncomine™ Breast cfDNA Assay (Thermo Fisher).

**Results:**

LP-WGS data demonstrated copy-number gains/losses in individual CTCs in regions including *FGFR1, JAK2* and *CDK6* in five patients, *ERBB2* amplification in two HER2-negative patients and *BRCA* loss in two patients. Seven of eight matched plasmas also had mutations in ctDNA in *PIK3CA, TP53, ESR1* and *KRAS* genes with mutant allele frequencies (MAF) ranging from 0.05 to 33.11%. Combining results from paired CTCs and ctDNA, clinically actionable targets were identified in all ten patients.

**Conclusion:**

This combined analysis of CTCs and ctDNA may offer a new approach for monitoring of disease progression and to direct therapy in patients with advanced MBC, at a time when they are coming towards the end of other treatment options.

## Introduction

Breast cancer (BC) is the most common cancer in women worldwide, accounting for more than half a million deaths annually [1]. Metastatic breast cancer (MBC) remains almost universally fatal, despite the introduction of improved targeted therapies [2]. In order to monitor the disease, clinicians have largely relied on blood tests (such as CA15-3) to determine the success of therapy, when tissue biopsy is not possible. Further, a single tissue biopsy may be unrepresentative of other metastatic deposits due to heterogeneity, and thus a more comprehensive characterisation of metastatic disease would be helpful for tailoring treatment decisions. The use of liquid biopsies, including circulating cell-free DNA (cfDNA) and circulating tumour cells (CTCs), has the advantages of being a minimally invasive approach that is suited to follow up over time by sequential sampling with the potential to guide precision medicine [3].

Numerous platforms are under investigation for isolation and characterisation of CTCs [4]; however, CellSearch^®^ (Menarini Silicon Biosystems), which enumerates CTCs of epithelial origin (CD45-, EpCAM+, and cytokeratins (CK) 8, 18+ and/or 19+ positive cells), remains the only FDA approved platform currently in clinical use. When detected, the number of these EpCAM-positive CTCs reflects both the effects of systemic therapy [5–7] and prognosis [8]. In the metastatic setting and in particular in treatment-refractory cancers, CTC profiling is an attractive approach to interrogate resistant cell populations in individual patients, in contrast to tissue-based analysis that can only provide information on the biopsied metastasis and deconvolution of cfDNA sequencing data at high depth [9–12]. Previous work has demonstrated mutation analysis of individual CTCs and pools of CTCs [13, 14]. We showed that plasma cfDNA and CTCs from the same blood sample provide complementary mutation information, but this was limited to the analysis of selected mutations across 50 cancer genes [3]. Recent mutation and copy-number profiling of 130 genes in archived CTCs showed that 61% of somatic copy-number aberrations (sCNAs) detected in CTCs were detected in tumour tissue whole exome sequencing (WES); in turn, tumour WES detected some alterations not detected in CTCs [15]. In this study, we introduce a more comprehensive global characterisation of copy-number aberrations in MBC through low-pass whole-genome sequencing (LP-WGS) of CTCs. We used CellSearch® followed by DEPArray™, a digital sorter that combines microfluidics and microelectronics to enable precise, image-based isolation of individual CTCs [16]. This workflow has been probed in several studies [13, 17–19]. Importantly, this comprehensive CTC LP-WGS approach has allowed us to identify clinically actionable targets to help guide treatment decisions in all patients analysed who are progressing at the time of blood sample.

## Materials and methods

### Ethics statement, patients and demographics

The study protocol was approved by the Imperial College Healthcare Tissue Bank (ICHTB) Ethics Committee (project reference number: 12/WA/0196). Blood sample collection was conducted in accordance with the Declaration of Helsinki. All patients gave written informed consent prior to participation. Female patients with radiologically-confirmed MBC, attending the breast oncology clinic at Charing Cross Hospital, London, were recruited for this study, as described previously [20]. All participants gave a single-EDTA blood sample and blood was also collected into a CellSave tube for CTC analysis [13].

The study population comprised 10 patients with MBC that all had successful recovery of CTCs by the CellSearch^®^/DEPArray™ workflow. Details of treatment(s) undergone by each patient at the time of blood sample collection were obtained from the Imperial College NHS electronic prescription system and where necessary, patient records. CT and MRI results confirmed by a consultant radiologist to determine disease status at the time of blood collection (Table 1). Data from scans closest to the time of blood sample collection (generally within 2 weeks) were used to evaluate disease response. Response to treatment was assessed using RECIST criteria [21] Five patients (2, 5, 6, 7 and 8) were included in a previous study analysing hot spot mutations in CTCs [13].

**Table 1:** Characteristics of the ten MBC patients analysed by CTC LP-WGS.

Patient ID	Sample ID	Primary tumour ER/PR/HER2 status			Treatment at the time of sample	Response at the time of sample	RIP	No. CTCs sequenced		Potential therapy indicated by CTS LP-WGS
1	–	ER +	PR +	HER2−	Letrozole, goserelin, palbociclib	Responding	Alive	3	+ pool	PARP inhibitor
2	Sample 1	ER +	PR +	HER2−	Tamoxifen	Progressing	RIP	4	+ pool	HER2 targeted therapy
	Sample 2				Tamoxifen	Progressing		11	+ pool	HER2 targeted therapy
3	–	ER +	PR +	HER2−	Unknown	Progressing	Alive	3		PARP inhibitor
4	–	ER +	NK	HER2−	Letrozole	Progressing	RIP	3	+ pool	PPM1D inhibitor
5	–	ER +	PR +	HER2−	Off treatment	Progressing	RIP	5	+ pool	FGFR inhibitor
6	–	ER−	PR−	HER2−	Off treatment	Progressing	RIP	11	+ pool	CDK4/6 inhibitor
7	–	ER +	PR +	HER2−	Epirubicin, goserelin	Responding	RIP	1		PARP inhibitor
8	–	ER +	PR +	HER2−	Capecitabine	Progressing	RIP	5	+ pool	JAK2 inhibitor
9	–	ER +	PR +	HER2−	Vinorelbine	Progressing	Alive	1	+ pool	CDK4/6 inhibitor
10	–	ER +	PR +	HER2−	Capecitabine	Progressing	RIP	3		FGFR inhibitor

Includes primary tumour characteristics, treatment and response status at the time of blood draw, and potential treatment options to target gene-specific sCNAs suggested by CTC LP-WGS. NK – not know, primary surgery at a different hospital.

### CTC recovery and whole-genome amplification (WGA)

CTC enrichment and enumeration were performed using the CellSearch^®^ System and the CellSearch^®^ Epithelial Cell Kit, and single cells, small pools of CTCs (range 8–23 cells per pool) and white blood cells (WBC) were recovered from the same CellSearch^®^ cassettes as the single cells (Supplementary Table 1) using the DEPArray™ system (all Menarini Silicon Biosystems, Bologna, Italy) according to the manufacturer’s protocols, as described previously [13]. Single cells and pools of cells were amplified using the Ampli1™ WGA kit, and the presence of amplified DNA was assessed by using the Ampli1™ QC Kit (Menarini Silicon Biosystems) and assessed by multiplex PCR as described previously [13]. Only samples with a genomic integrity index (GII) ≥ 2 were analysed.

### Low-pass whole-genome sequencing

LP-WGS was performed using the Ampli1™ LowPass Kit for the Ion Torrent (Menarini Silicon Biosystems) workflow as described previously [22]. Libraries were pooled, and template preparation was performed using the Ion Chef^TM^, loaded onto an Ion 530™ and sequenced using the S5 platform (Thermo Fisher Scientific). All samples with <200,000 reads were excluded from the analysis. Copy-number aberrations were identified using the MSBiosuite software platform (Menarini Silicon Biosystems) and R (version 4.0.1) [23]. All results showing a copy-number value ≥4 were noted as amplified and ≤1 as loss. The GISTIC algorithm [24] was applied to the binned data from the MSBiosuite output.

For a visual representation, all results generated were filtered through Cosmic Cancer Gene Census [25] and Cancer Genome Interpreter (CGI) [26] databases to create a list of relevant, targetable cancer genes. Only potentially actionable alterations, of clinical importance are presented as results.

### Oncomine breast cfDNA assay

Total cfDNA was isolated from 4 ml of plasma using the MagMAX Cell-free DNA Isolation Kit (Thermo Fisher Scientific) according to the manufacturer’s instructions. Genomic DNA (gDNA) was isolated from 200 μl buffy coat (for germline DNA (gDNA)) as described previously [13].

Library reactions were set up on the Ion Chef™ system according to the manufacturer’s protocol, using the Oncomine™ Breast cfDNA v1 Assay and run on a 540 chip on the Ion S5 XL sequencing platform. Alignment of sequencing raw data was performed by the Torrent Suite Software version v 5.12 (all Thermo Fisher Scientific). All high-confidence variant calls (those with an allele molecular coverage of >2 and Allele Mol Freq (MAF%) > the LOD for each variant) were reviewed manually using the Integrated Genomics Viewer (IGV) package (v2.3.25) [27] by two observers.

## Results

**Fig. 1:**
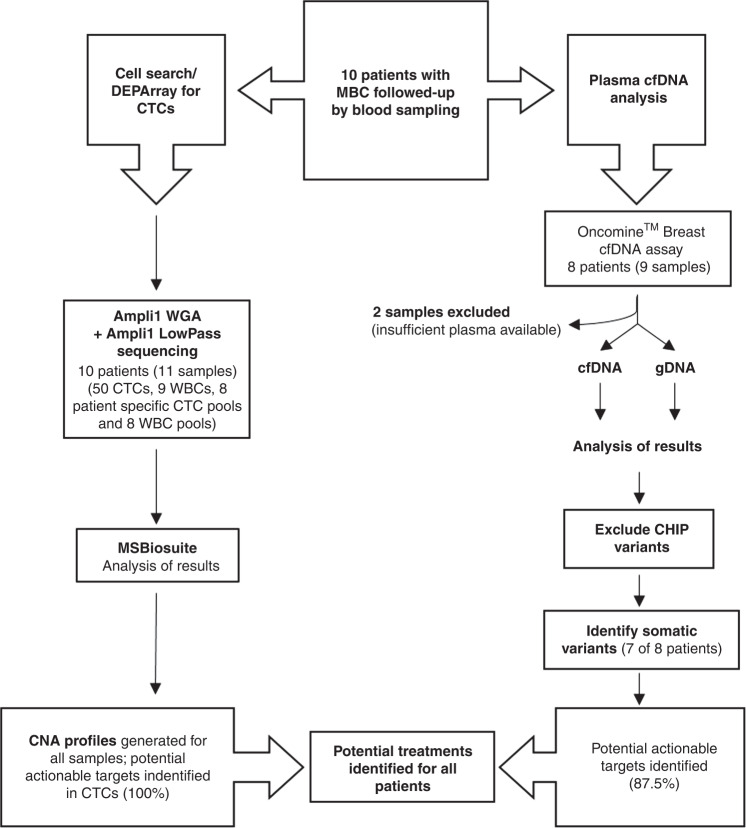
Study flow diagram. Showing the total number of patients, samples and single cells/pools included in the study, as well as the number of excluded patients or samples, and the reason behind the exclusion.

Ten patients with radiologically-confirmed MBC, were investigated as part of this study, all of whom had >20 CTCs detected by CellSearch^®^ from a single 7.5 ml blood draw. Eight patients were progressing on treatment at the time of blood sampling, and two were responding to their treatment. Eleven blood samples from the ten patients were analysed with the CellSearch^®^ and DEPArray™ workflow. Of these, 59 single cells (50 CTCs and 9 WBCs) and 16 cell pools (8 paired CTC pools and 8 WBC pools) across 11 blood samples from the 10 patients generated successful LP-WGS results for sCNA analysis (>200,000 reads) (Fig. 1, Table 1 and Supplementary Table 1).

### Low-pass whole-genome sequencing reveals clinically important targets in individual CTCs

**Fig. 2:**
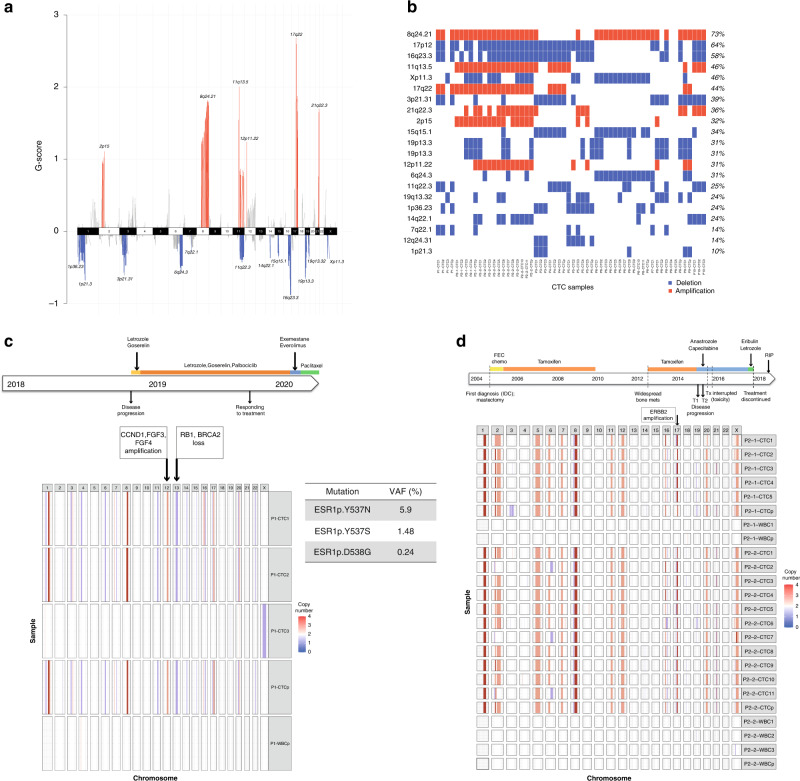
LP-WGS identifies significant copy-number regions in CTCs. **a** GISTIC plot of genomic regions with significant CN gain (red) or loss (blue) (FDR *q*-value <0.01) from XX CTCs from ten patients. The 21 autosomes and Chromosome X are shown on the *X* axis, and a G-score on the *Y* axis where each aberration is assigned a value that considers the amplitude as well as the frequency of its occurrence across the CTC cohort. False Discovery Rate *q*-values are then calculated for the aberrant regions, and regions with *q*-values below 0.01 were considered significant. Regions in grey fall below the significance threshold. **b** Oncoplot of the 21 significant regions identified from the GISITC analysis, detailing which aberrations are present in CTCs from the ten patients. Red is associated with an increase in copy number (gain/amplification) and blue a loss/deletion. **c** Treatment timeline for Patient 1, highlighting treatment received and the time of blood sample. A CN spectrum plot for all CTCs and the WBCp for this patient reveal possible actionable copy-number aberrations consistent in CTC1, 2 and the CTCp on chromosome 12 (amplification of *CCND1, FGF3, FGF4*) and chromosome 13 (loss of *RB1* and *BRCA2*). Oncomine analysis of plasma cfDNA identified *ESR1* mutations. **d** Treatment timeline for Patient 2, highlighting treatment received the time when the two blood samples were obtained. A CN spectrum plot for all CTCs show homogenous genomic profiles from time points 1 and 2 with amplifications on chromosomes 1, 8, 12 and 17 consistent throughout all CTC samples identified. The single WBC and WBC pools show a flat genomic profile with no aberrations detected. CN profiles of CTCs from this patient reveal a region of amplification that contains the *ERBB2* gene not indicated from the primary tumour suggesting this has evolved through the course of the disease and could have provided a potential targeted treatment option.

To investigate significant regions of recurrent amplification/gain and loss regions in CTCs, the GISTIC algorithm was applied to the genome-wide copy-number data generated for each individual CTC and CTC pool and the cancer genome interpreter (25) was used to identify recurrent copy-number regions with an associated cancer gene. Across the 10 patients, 21 regions showing significant recurrent amplifications or deletions (*q*-value <0.05) were observed, including 6 regions of amplifications and 15 regions of deletion (Fig. 2a, b and Supplementary Table 2). The most commonly found recurrent region was 8p24.21 (chr8: 91,305,140–146,364,022, 44 samples, 76% of CTC cohort), containing the MYC oncogene, whilst the most frequent recurrent deletion was 17p12 containing known tumour suppressor genes *TP53, MAP2K4* (chr17: 6,802,327–27,406,782, 39 samples, 67% of CTC cohort). Other regions of note include amplification of 11q13.5 (*CCND1, FGF3, FGF4*), 17q22 (*PPM1D*), 2p15 (*XPO1*), 12p11.22 and 21q22.3 and deletion of 16q23.3 (*FANCA*), 3p21.3 (*BAP1, SETD2, PBRM1*), 19p12.3 (*STK11*) and 11q22.3 (*ATM*) (Fig. 2a, b and Supplementary Table 2).

We sought to establish if any additional treatment could be proposed based on the amplification and deletion events called in CTCs. We mapped actionable genes from the cancer genome interpreter to each CTC genomic profile (Supplementary Tables 3 and 4). Two of the ten patients were responding to their treatment at the time of blood sample (patient IDs 1 and 7; Table 1). Patient ID 1 was responding to Letrozole, Goserelin, and Palbociclib; three individual CTCs and a CTC pool were sequenced successfully from this patient. Two of the individual CTCs (CTC1 and CTC2, Fig. 2c) and the CTC pool had homogenous copy-number profiles, and included amplification on 1q and 8q (*MYC*), 11q (*CCND1, FGF3, FGF4*) and allelic loss of regions including several cancer genes on 3p (BAP1), 13q(*RB1, BRCA2*) and 17p (*MAP2K4*). The third individual CTC (CTC3) had lost one copy of the X chromosome, but otherwise had a flat profile similar to the white blood cell pool (Fig. 2c, Supplementary Table 3 and Supplementary Fig. 1), despite having all phenotypic characteristics of a CellSearch^®^ CTC (EpCAM+/CK+/CD45−). Analysis of the individual CTC segments and CTC pool (Supplementary Table 3) revealed loss of *RB1* (13q) in this patient, which suggested that she may develop resistance to her current treatment, palbociclib, as was the case confirmed by her notes. Further, LP-WGS revealed novel genetic changes that would have suggested other potential treatment options, such as PARP inhibitors for *BRCA2* loss (Table 1 and Fig. 2c). Patient ID 7, who was receiving epirubicin and goserelin at the time of blood draw, had only a single CTC recovered. However, of note, loss of 13q including *BRCA1* in this CTC indicated that a PARP inhibitor could potentially have been offered as an alternative treatment (Supplementary Table 3).

Of the eight patients who were progressing at the time of blood sampling, patient two had two blood samples taken a month apart (Time points 2.1 and 2.2). A total of 15 individual CTCs across the two blood samples were analysed with a CTC pool from each sample. In our previous study, no somatic mutations were detected across hot spot mutations in 50 cancer genes in any CTCs, or matched plasma cfDNA samples from this patient [13]. However, here LP-WGS identified sCNAs in all CTCs, notably segments with copy-number gain, including *CCND1, FGFR1*, *ERBB2 and PPM1D*. Although the primary tumour was HER2-negative, amplification of a segment including the *ERBB2* gene was detected in 12/15 CTCs analysed across the 2 blood samples and both CTC pools (Fig. 2d and Supplementary Tables 3 and 4), suggesting the patient had acquired *ERBB2* amplification during adjuvant therapy and might therefore have responded to Herceptin if it had been administered. The other seven patients that had progressive disease at the time of blood draw, also had multiple actionable targets identified by LP-WGS of individual CTCs (Table 1 and Supplementary Tables 3 and 4). For example, patient 5, who was off treatment at the time the blood sample was taken, had a copy-gained segment containing suggesting *FGFR1* amplification and another segment indicating *BRCA* gene loss by CTC LP-WGS.

### Individual CTCs and cfDNA can provide complementary information to guide therapy

We also performed mutation profiling in nine plasma cfDNA samples from eight of the ten patients using the Oncomine™ Breast cfDNA Assay (Thermo Fisher) and compared results with paired gDNA to eliminate the interference of any somatic mutations in clonal hematopoiesis (CHIP) [28–30]. No plasma was available for two patients. No variants were detected in the two serial samples from patient 2. The seven other patient samples had one or more somatic variants detected in *PIK3CA, TP53, ESR1* and *KRAS* genes with mutant allele frequencies (MAF) ranging from 0.05 to 33.11% (Supplementary Table 5). Of note, five patients had mutations in *ESR1*, indicating resistance to endocrine therapy and we found no mutations across all eight gDNA samples.

## Discussion

This study represents a detailed characterisation of sCNAs in multiple individual CTCs obtained from ten patients with MBC using the CellSearch^®^/DEPArray™/Ampli1™ LP-WGS workflow. In each of the ten patients, results from CTC LP-WGS highlight clinically relevant sCNAs including cancer genes that could have guided changes to treatment. Frequently amplified regions include key driver genes *CCND1, FGFR, ERBB2, JAK2* and *ESR1*. *FGFR1, CCND1* and *ESR1* gene amplifications have been found in CTCs from endocrine therapy-resistant HR + MBC [15] while *CCND1* amplification was also present in an ER+ and a triple-negative MBC patient with brain metastasis [31]. The most prominent deleted regions contain genes regulating chromatin accessibility (*STK11, SETD2, SUZ12, PBRM1, KDM6A, BAP1, ARID1A*) and DNA repair (*BRCA1/2, APOBEC3B*). Moreover, our results show heterogeneity in copy-number patterns across single CTCs, as has been reported previously. Ni et al. [28] showed that the copy-number patterns of CTCs are reproducible within CTCs of the same patient and in different patients with the same breast cancer subtype, whilst others have reported mixed results, with almost a third of the patients showing heterogeneous copy-number patterns [17].

Other studies have either compared CTCs and plasma in breast cancer patients but only carried out CellSearch^®^ CTC enumeration [32] or compared CTCs and tissue DNA, but performed an amplicon-based NGS panel targeting a limited set of genes [15]. Whole-genome copy-number analysis of single CTCs was used in breast cancer patients with liver [33] or brain metastasis [31]. However, CNA profiles of individual CTCs have been not been utilised in MBC as potential biomarkers for clinical stratification of patients as in small cell lung cancer (SCLC), where they have been shown to be capable of distinguishing chemosensitive from the chemorefractory disease [17, 34].

A previous array comparative hybridisation study [35] showed that CTCs obtained from patients with MBC fell into three main groups based on the extent of genomic aberrations and the presence of large chromosomal imbalances. Another study demonstrated that CTCs had different degrees of sCNA burden based on the time point and subtype examined in early-stage BC [36]. Our data show that CTCs in MBC are characterised by genomic heterogeneity, suggesting the presence of circulating pools of heterogeneous sub-clones that are presumably responsible for the emergence of resistance to therapies, which is a feature commonly seen in patients with MBC. Advances in technologies for analyses of CTCs at the single-cell level have facilitated the characterisation and the monitoring of dynamic changes in tumour heterogeneity [37].

Here, the combination of CTC LP-WGS with mutation analysis of plasma cfDNA identified additional information towards guiding therapy. For example, patient ID 1 (ER+/HER2−), was undergoing aromatase inhibitor (AI) treatment at the time of blood sampling. The emergence of polyclonal *ESR1* mutations (p.Y537N, p.Y537S and p.D538G) in her plasma cfDNA indicated emerging resistance to AI treatment that could have been used to change treatment. Previously, we and others demonstrated that targeted mutational analysis of single CTCs may enable monitoring of the metastatic burden for clinical decision-making [13, 37]. Our direct approach in the current study showed important advantages since LP-WGS has allowed an unbiased genome-wide survey of sCNAs, and highlighted that the analysis of individual CTCs is necessary to determine the aggregate genetic signatures in CTCs. As highlighted here, technical difficulties remain regarding the analysis of single cells recovered from blood, as a significant proportion of samples either failed the post-recovery sample QC or failed to generate sufficient sequence data for genomic interrogation. Despite these challenges, this approach provided useful information in all patients where CTC LP-WGC was achieved.

In terms of clinical application, most of the ten MBC patients had progressive drug-resistant metastatic breast cancer and nearly all patients died within 6 months of the blood sample being taken. However, had liquid biopsy analysis of CTCs and plasma cfDNA been available to aid clinical decisions, all patients could potentially have been offered an alternative therapy (Table 1). As the samples were obtained (in most cases) within a few months of the death, we therefore do not yet know if CTCs are able to provide similarly useful information for clinicians earlier in the disease. However, due to the difficulty and potential morbidity inherent in obtaining sequential tissue biopsies from metastatic sites, clinicians are frequently unable to determine the optimal treatment for MBC patients. As more targeted treatments become available, it will be important to obtain maximal genomic information before starting costly and sometimes toxic therapies that can adversely affect their quality of life. Our study offers hope to rectify this situation by enabling a more rational selection of therapies. Finally, all except one patient had ER-positive breast cancer; therefore, we are not certain that triple-negative breast cancer patients will also display actionable mutations in CTCs, but current studies are addressing this issue. Since current clinical practice in the treatment of ER-positive breast cancer is to exhaust all non-chemotherapy therapies prior to switching to chemotherapy, CTC LP-WGS patients could help identify those patients that acquire *ERBB2* amplification and herald a switch to HER2-directed treatments before more toxic cytotoxic chemotherapy.

In conclusion, this study highlights the potential of CTC LP-WGS for identifying clinically important targets to inform treatment decisions in patients with advanced MBC at a time when they were coming towards the end of other treatment options. In each patient, one or more targets was identified that could have heralded a change in treatment, including HER2 and FGFR-directed therapies, JAK2 and PARP inhibitors. The work requires further evaluation in a prospective study but may offer a new approach to managing treatment decisions in MBC for those patients with detectable CTCs.

## Supplementary information

Supplementary Figure 1

Supplementary Tables

checklist

## Data Availability

The datasets used and/or analysed during the current study are available from the corresponding author on reasonable request. All data analysed during this study are included in this published article (and its supplementary information files).

## References

[1] Siegel RL, Miller KD, Jemal A (2018). Cancer statistics. CA Cancer J Clin.

[2] Ali S, Buluwela L, Coombes RC (2011). Antiestrogens and their therapeutic applications in breast cancer and other diseases. Annu Rev Med.

[3] Bardelli A, Pantel K (2017). Liquid biopsies, what we do not know (yet). Cancer Cell.

[4] Rushton AJ, Nteliopoulos G, Shaw JA, Coombes RC. A review of circulating tumour cell enrichment technologies. Cancers. 2021;13:970. 10.3390/cancers13050970 PMC7956528 33652649

[5] Rack B, Schindlbeck C, Juckstock J, Andergassen U, Hepp P, Zwingers T, et al. Circulating tumor cells predict survival in early average-to-high risk breast cancer patients. J Natl Cancer Inst. 2014;106:dju066. 10.1093/jnci/dju066 PMC4112925 24832787

[6] de Bono JS, Scher HI, Montgomery RB, Parker C, Miller MC, Tissing H (2008). Circulating tumor cells predict survival benefit from treatment in metastatic castration-resistant prostate cancer. Clin Cancer Res.

[7] Smith BM, Slade MJ, English J, Graham H, Luchtenborg M, Sinnett HD (2000). Response of circulating tumor cells to systemic therapy in patients with metastatic breast cancer: comparison of quantitative polymerase chain reaction and immunocytochemical techniques. J Clin Oncol.

[8] Cristofanilli M, Budd GT, Ellis MJ, Stopeck A, Matera J, Miller MC (2004). Circulating tumor cells, disease progression, and survival in metastatic breast cancer. N. Engl J Med.

[9] Jamal-Hanjani M, Wilson GA, McGranahan N, Birkbak NJ, Watkins TBK, Veeriah S (2017). Tracking the evolution of non-small-cell lung cancer. N. Engl J Med.

[10] Abbosh C, Birkbak NJ, Wilson GA, Jamal-Hanjani M, Constantin T, Salari R (2017). Phylogenetic ctDNA analysis depicts early-stage lung cancer evolution. Nature.

[11] Robinson DR, Wu YM, Vats P, Su F, Lonigro RJ, Cao X (2013). Activating ESR1 mutations in hormone-resistant metastatic breast cancer. Nat Genet.

[12] Roychowdhury S, Iyer MK, Robinson DR, Lonigro RJ, Wu YM, Cao X (2011). Personalized oncology through integrative high-throughput sequencing: a pilot study. Sci Transl Med.

[13] Shaw JA, Guttery DS, Hills A, Fernandez-Garcia D, Page K, Rosales BM (2017). Mutation analysis of cell-free DNA and single circulating tumor cells in metastatic breast cancer patients with high circulating tumor cell counts. Clin Cancer Res.

[14] Paolillo C, Mu Z, Rossi G, Schiewer MJ, Nguyen T, Austin L (2017). Detection of activating estrogen receptor gene (ESR1) mutations in single circulating tumor cells. Clin Cancer Res.

[15] Paoletti C, Cani AK, Larios JM, Hovelson DH, Aung K, Darga EP (2018). Comprehensive mutation and copy number profiling in archived circulating breast cancer tumor cells documents heterogeneous resistance mechanisms. Cancer Res.

[16] Di Trapani M, Manaresi N, Medoro G (2018). DEPArray system: an automatic image-based sorter for isolation of pure circulating tumor cells. Cytom A.

[17] Carter L, Rothwell DG, Mesquita B, Smowton C, Leong HS, Fernandez-Gutierrez F (2017). Molecular analysis of circulating tumor cells identifies distinct copy-number profiles in patients with chemosensitive and chemorefractory small-cell lung cancer. Nat Med.

[18] De Luca F, Rotunno G, Salvianti F, Galardi F, Pestrin M, Gabellini S (2016). Mutational analysis of single circulating tumor cells by next generation sequencing in metastatic breast cancer. Oncotarget.

[19] Hodgkinson CL, Morrow CJ, Li Y, Metcalf RL, Rothwell DG, Trapani F (2014). Tumorigenicity and genetic profiling of circulating tumor cells in small-cell lung cancer. Nat Med.

[20] Fernandez-Garcia D, Hills A, Page K, Hastings RK, Toghill B, Goddard KS (2019). Plasma cell-free DNA (cfDNA) as a predictive and prognostic marker in patients with metastatic breast cancer. Breast Cancer Res.

[21] Eisenhauer EA, Therasse P, Bogaerts J, Schwartz LH, Sargent D, Ford R (2009). New response evaluation criteria in solid tumours: revised RECIST guideline (version 1.1). Eur J Cancer.

[22] Ferrarini A, Forcato C, Buson G, Tononi P, Del Monaco V, Terracciano M (2018). A streamlined workflow for single-cells genome-wide copy-number profiling by low-pass sequencing of LM-PCR whole-genome amplification products. PLoS ONE.

[23] R_Core_Team. R: a language and environment for statistical computing. Vienna, Austria: R Foundation for Statistical Computing; 2020.

[24] Mermel CH, Schumacher SE, Hill B, Meyerson ML, Beroukhim R, Getz G (2011). GISTIC2.0 facilitates sensitive and confident localization of the targets of focal somatic copy-number alteration in human cancers. Genome Biol.

[25] Sondka Z, Bamford S, Cole CG, Ward SA, Dunham I, Forbes SA (2018). The COSMIC Cancer Gene Census: describing genetic dysfunction across all human cancers. Nat Rev Cancer.

[26] Tamborero D, Rubio-Perez C, Deu-Pons J, Schroeder MP, Vivancos A, Rovira A (2018). Cancer Genome Interpreter annotates the biological and clinical relevance of tumor alterations. Genome Med.

[27] Robinson JT, Thorvaldsdottir H, Winckler W, Guttman M, Lander ES, Getz G (2011). Integrative genomics viewer. Nat Biotechnol.

[28] Ni X, Zhuo M, Su Z, Duan J, Gao Y, Wang Z (2013). Reproducible copy number variation patterns among single circulating tumor cells of lung cancer patients. Proc Natl Acad Sci USA.

[29] Fribbens C, Garcia Murillas I, Beaney M, Hrebien S, O’Leary B, Kilburn L (2018). Tracking evolution of aromatase inhibitor resistance with circulating tumour DNA analysis in metastatic breast cancer. Ann Oncol.

[30] Shibayama T, Low SK, Ono M, Kobayashi T, Kobayashi K, Fukada I (2020). Clinical significance of gene mutation in ctDNA analysis for hormone receptor-positive metastatic breast cancer. Breast Cancer Res Treat.

[31] Riebensahm C, Joosse SA, Mohme M, Hanssen A, Matschke J, Goy Y (2019). Clonality of circulating tumor cells in breast cancer brain metastasis patients. Breast Cancer Res.

[32] Davis AA, Zhang Q, Gerratana L, Shah AN, Zhan Y, Qiang W (2019). Association of a novel circulating tumor DNA next-generating sequencing platform with circulating tumor cells (CTCs) and CTC clusters in metastatic breast cancer. Breast Cancer Res.

[33] Zou L, Imani S, Maghsoudloo M, Shasaltaneh MD, Gao L, Zhou J (2020). Genomewide copy number analysis of circulating tumor cells in breast cancer patients with liver metastasis. Oncol Rep..

[34] Su Z, Wang Z, Ni X, Duan J, Gao Y, Zhuo M (2019). Inferring the evolution and progression of small-cell lung cancer by single-cell sequencing of circulating tumor cells. Clin Cancer Res.

[35] Magbanua MJM, Rugo HS, Wolf DM, Hauranieh L, Roy R, Pendyala P, et al. Expanded genomic profiling of circulating tumor cells in metastatic breast cancer patients to assess biomarker status and biology over time (CALGB 40502 and CALGB 40503, Alliance). Clin Cancer Res. 2018;24:1486–99. 10.1158/1078-0432.CCR-17-2312 PMC5856614 29311117

[36] Rossi T, Gallerani G, Angeli D, Cocchi C, Bandini E, Fici P, et al. Single-cell NGS-based analysis of copy number alterations reveals new insights in circulating tumor cells persistence in early-stage breast cancer. Cancers. 2020;12:2490. 10.3390/cancers12092490 PMC7565733 32887501

[37] Keller L, Pantel K (2019). Unravelling tumour heterogeneity by single-cell profiling of circulating tumour cells. Nat Rev Cancer.

