# Supplementary material for: Shallow WGS of individual CTCs identifies actionable targets for informing treatment decisions in metastatic breast cancer
Source: Br J Cancer. 2022 Sep 10;127(10):1858–64. doi: 10.1038/s41416-022-01962-9 (PMC9643413; doi:10.1038/s41416-022-01962-9)
Supplement: Supplementary file 1 — Supplementary Figure 1 [file 41416_2022_1962_MOESM1_ESM.pdf]

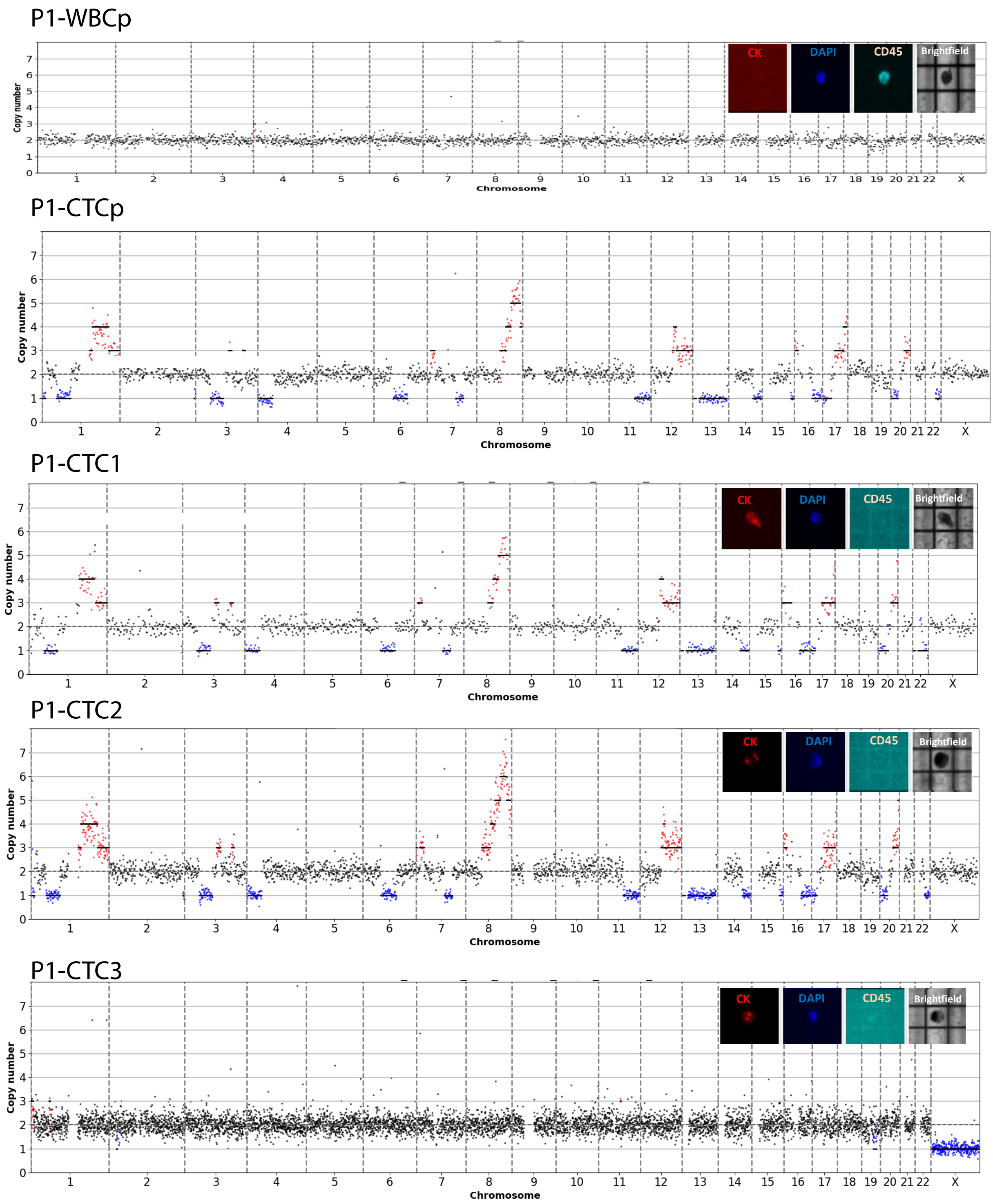

Supplementary Figure 1. Patient 1 CTC Genome wide profiles were generated using MSBiosuite. P1-WBCp (white blood cell pool) shows a normal profile with no genomic aberrations. DEPArray™ images from two of the single WBCs included in this pool P1-CTCp (CTC pool) shows a genome wide profile comparable to CTC 1 and CTC 2, with very little heterogeneity. However even though by DEP array CTC 3 is suggested to be a CTC the genomic profile is flat showing only loss of chromosome X.
